# Extracellular microRNA-21 and microRNA-26a increase in body fluids from rats with antigen induced pulmonary inflammation and children with recurrent wheezing

**DOI:** 10.1186/s12890-016-0216-2

**Published:** 2016-04-14

**Authors:** Congshan Jiang, Hongchuan Yu, Qingzhu Sun, Wenhua Zhu, Jing Xu, Ning Gao, Rui Zhang, Li Liu, Xiaoying Wu, Xudong Yang, Liesu Meng, Shemin Lu

**Affiliations:** Department of Biochemistry and Molecular Biology, School of Basic Medical Sciences, Xi’an Jiaotong University Health Science Center, West Yanta Road No.76, Xi’an, Shaanxi PR China; Key Laboratory of Environment and Genes Related to Diseases (Xi’an Jiaotong University), Ministry of Education, Xi’an, Shaanxi PR China; Department of Respiratory Medicine, Xi’an Children Hospital, Xi’an, Shaanxi PR China; Department of Clinical Laboratory, the Second Affiliated Hospital of Xi’an Jiaotong University, Xi’an, Shaanxi PR China; Department of Bone and Joint Diseases, Xi’an Honghui Hospital, Xi’an, Shaanxi PR China

**Keywords:** Childhood wheezing, Antigen induced pulmonary inflammation, Animal model, Extracellular miRNA

## Abstract

**Background:**

This study aims to find out whether extracellular miRNAs is implicated in recurrent childhood wheezing with asthmatic risk.

**Methods:**

One hundred and forty children of Chinese Han population were recruited for this study. Plasma and intracellular miRNAs from children with recurrent wheezing and rats with antigen induced pulmonary inflammation (AIPI) were detected by using reverse transcription-quantitative PCR. Differential leukocytes in blood were automatically counted. Total IgE was detected by enzyme-linked immunosorbent assay. Clinical implication in diagnosis was evaluated using receiver operating characteristic curves.

**Results:**

The increase of plasma miR-21 and miR-26a was screened out from 11 candidate miRNAs and validated in wheezing children. The level of expression for both miRNAs were comparable in different age and gender. Plasma miR-21 was more preferable to miR-26a and total IgE for diagnosis. Plasma miR-21 and miR-26a levels were not significantly correlated with various leukocyte counts or miRNA expression in blood cells. In acute and chronic AIPI rats, miR-21 levels increased in both plasma and lavaged lung compared with control. Moreover, circulating miR-21 and miR-26a levels were highly positively correlated with infiltrated cell counts in bronchoalveolar lavage fluid of AIPI rats.

**Conclusions:**

Circulating miR-21 and miR-26a increase in wheezing children and AIPI rats. This not only manifests their strong clinical implication in recurrent childhood wheezing with asthma risk, but also provides novel insights into the role of extracellular miRNAs during development of airway inflammation and recurrent wheezing.

**Electronic supplementary material:**

The online version of this article (doi:10.1186/s12890-016-0216-2) contains supplementary material, which is available to authorized users.

## Background

Pediatric bronchial asthma is one of the most commonest chronic airway inflammatory disorders and leads to airway hyperreactivity (AHR) triggering recurrent episodes of wheeze, shortness of breath, chest tightness and cough [1]. Without proper treatment, the asthmatic children may suffer with a progression or remission of symptoms for the rest of life. Early diagnosis, prevention and drug intervention are thus very critical. Unfortunately, objective methods of detection such as physical examination of lung function and airway inflammation are unavailable for children under 5 years old. Lack of effective detection methods makes the present diagnosis for pediatric bronchial asthma difficult.

Childhood recurrent (three or more episodes) wheezing as a hazardous sign has long been considered as subjective characteristic of much controversy. A considerable number of children experience wheezing when they suffer from lower respiratory infection, and many of these children later develop pediatric asthma after school age. Recurrent wheezing is often considered as an important risk factor for developing asthma [2]. In addition, familial asthma was found as risk factors in infants during their first year of life [3]. There is an inextricable link between pediatric asthma and recurrent wheezing during lower respiratory infection. Therefore, we aim to identify biomarkers for pediatric wheezing which could be further developed for asthma diagnosis in future.

Different studies have been performed to identify and characterize non-invasive diagnostic biomarkers in blood, sputum and urine samples from patients [4]. For instance, multiple allergen-specific IgE in blood has been recommended as a biomarker for asthma. The advantage of this is the ease of sample collection. The disadvantage of using serum IgE as a biomarker is that, IgE level can be influenced by age, gender and ethnics [5]. Clinical tests have suggested that exhaled NO can serve as an indicator for airway inflammation, although this method is not very specific [6]. Some exhaled biomarkers such as fractional exhaled nitric oxide (FeNO) have been widely used to help evaluate disease management in pediatric lung diseases, and considered to be helpful in predicting onset and exacerbations of lung diseases [7]. In future, the identification of more sensitive markers will be very important for better diagnosis and management of pediatric lung disease such as pediatric asthma and pediatric wheezing.

In recent years, circulating miRNAs as molecular biomarkers of diseases have aroused great attention. Circulating miRNAs were first found in serum of patients with tumor in 2008 and have been believed to be extremely stable even under harsh conditions including heat, extreme pH, long time storage and repeated freezing and thawing. The extreme stability of circulating miRNA displays is a natural advantage as a biomarker. In healthy individuals, the existence of circulating and intracellular miRNAs in blood cells is homogeneous [8], but can be changed in pathological conditions [9]. Several miRNAs have been found to play important roles in asthmatic animal organs and human pulmonary cells (Additional file 1: Table S1) [10–21]. The circulating miRNAs in body fluids may represent an undiscovered treasure of noninvasive markers for pediatric asthma.

In this study, we aim to investigate whether there is a differential expression of miRNAs in plasma of wheezing children, and evaluate their clinical potential in recurrent wheezing. These circulating miRNAs are then further studied in animals with antigen induced pulmonary inflammatory to investigate whether and how they might be involoved in the development of airway inflammation.

## Methods

### Design of the study

The main objective of this study is to identify suitable circulating miRNAs in wheezing children which can be used to help diagnose pediatric bronchial asthma in future. Both control and wheezing children were recruited for plasma miRNA detection. MiRNA were detected using two patient samples. Firstly, the expression level of 11 miRNAs known to be involved in asthma pathology were studied using samples form a small cohort of patients. Then another validation test was performed, and the theoretical minimum sample volume of the validation sample was calculated according to the results from screening test. Stability and diagnosis potential of these miRNAs was evaluated, and their expression level were further detected in plasma, BALF, lung and spleens from rats with pulmonary inflammation.

The initial search for candidate miRNAs in wheezing children were performed on samples from 20 wheezing + LRI children and 20 lower respiratory infection (LRI) controls (gender-matched and randomly selected) to search candidate miRNAs in wheezing children. Age of the patients and controls were compared and there was no significance difference (*p* > 0.10) between the two groups. Selected miRNAs were subsequently validated on a cohort of 70 wheezing + LRI patients, 35 indifferent controls, and 35 LRI controls. The subjects were further divided into infants (0–2 years old), preschool children (2–5 years old) and school age children (5–12 years old) with a fixed proportion. Male/female ratios were similar/comparable in these three groups, and age distribution was very similar. Balance of patients’ ages in validation sample was analyzed and there was no difference in the age of patients among three groups or between any two groups (*p* > 0.05) (Additional file 1: Figure S1).

### Patients’ information

#### Patient recruitment

One hundred and forty children of Chinese Han population were recruited from Department of Respiratory Medicine in Xi’an Children Hospital. Since childhood wheezing in practice is commonly complicated with lower respiratory infection (LRI), the children chosen as subjects were specifically diagnosed as wheezing with clinical manifestations of bronchopneumonia or bronchitis (called “wheezing + LRI” group). Pediatric bronchial asthma was diagnosed according to the criteria of “Global strategy for asthma management and prevention” [22]. Patients were recruited when they were first diagnosed with the symptom, and blood samples were also collected during the examination of physicians before any medical treatment. For both the study and control groups, the children were less than 12 years old. All subjects were not diagnosed with congenital heart diseases, intestinal infection, upper respiratory diseases, complications with liver or kidney injury or Kawasaki disease and any type of malignancy.

#### Clinical characteristics of patients

For wheezing + LRI group, the children chosen as subjects were specifically diagnosed as wheezing and also had clinical manifestations of bronchopneumonia or bronchitis. The study group has clinical manifestations of recurrent wheezing (three or more episodes of wheeze). Children with wheezing caused by abnormal airway development, airway foreign body, tumor and respiratory tuberculosis were excluded. The children were also known to not have any allergy attack in the 6 months prior to sample collection. The children did not take medications such as inhaled corticosteroid, maintenance oral prednisolone, long-acting β-agonist (LABA), leukotriene receptor antagonist and theophylline within 6 months prior to the collection of samples.

Clinical characteristics of patients are described in Table 1. Pulmonary function of wheezing children with asthmatic diagnosis was measured and analyzed by automatic spirometer (Jaeger company). In vitro allergen tests were performed using Euroline Atopy kits (Euroimmun AG company), a membrane based sandwish ELISA assay. The membrane was coated with 21 antigen specific human IgE antibody. Mouse anti-human IgE antibody labeled with AP was used as secondary antibody for detection of positive immune response in plasma samples.

**Table 1:** Clinical characteristics of patients

Characteristics	Individuals with recurrent wheezing		Wheezing individuals with asthmatic diagnosis
Groups	Infancy (0–2 years old)	Pre-school children (2–5 years old)	School children (5–12 years old)
Age on first wheezing attack (years old)	0.42 (0.25–0.63)	1.63 (1–2.5)	4 (3–5.17)
Duration of symptoms (years)	0.33 (0.25–0.92)	1 (0.63–2)	2 (1–3)
Hospitalization times due to wheezing attack	2 (1–2)	2 (2–2.25)	3 (2–4)
FEV_1_			
FEV_1_ predicted (L)	-		1.69 (1.12–2.3)
FEV_1_ pre-treatment (L)	-		1.23 (0.8–1.38)
FEV_1_ pre-treatment (% predicted)	-		72.4 (67.3–76.1)
FEV_1_/FVC ratio			
FEV_1_/FVC ratio predicted	-		0.8512 (0.8438–0.8539)
FEV_1_/FVC ratio pre-treatment	-		0.7008 (0.6828–0.7059)
FEV_1_/FVC ratio pre-treatment (% predicted)	-		81.7 (80.1–83.4)
In vitro allergen test	-		Various allergens are confirmed such as soybean, mite collection, codfish, lobster, fan-shell, shrimp and house dust.

Values in Table 1 are presented as medians (IQRs), *IQRs* interquartile ranges. Medications in the table refer to drug treatment before sample collections. In vitro allergen test and detection of lung function such as forced expiratory volume in one second (FEV_1_) and forced vital capacity (FVC) are not available for children less than 5 years old

#### Sample composition

Two gender- and age-matched control groups were included in this study. Children in the first control group named “LRI control” suffered from bronchopneumonia or bronchitis, and children in the second group named “indifferent control” were free from any respiratory symptoms. Each group consists of half infants and half children with almost identical proportion for gender and age (Table 2).

**Table 2:** Composition of sample for the validation test

Group	Cases	Infancy (0 < × ≤ 2)	Preschool (2 < × ≤ 5)	School age (5 < × ≤ 12)	Male/Female	Age
Indifferent control	35	17	9	9	26/9	4.1 ± 0.6
LRI control	35	17	9	9	26/9	3.5 ± 0.6
Wheezing + LRI	70	35	18	17	53/17	3.3 ± 0.4

LRI refers to lower respiratory infection, and quantitative data are shown as mean ± SEM

#### Procedures and ethics

The research involving human subjects is in accordance with the Declaration of Helsinki (revised in 2013). This study were approved by both Xi’an Children Hospital and Xi’an Jiaotong University Ethics Committee. Written informed consents were obtained from parents as well as guardians of the participants.

#### Determination of sample volume

The number of subjects per group was calculated according to the results from screening test. The formula $n={\frac{2{\sigma}^2\left({t}_{\alpha }+{t}_{\beta}\right)}{{\left({\mu}_1-{\mu}_2\right)}^2}}^2$ was applied in calculation for the theoretical minimum sample volume (Additional file 1: Table S2), in which α = 0.05, β = 0.2, t_α_ = 1.96, t_β_ = 0.842, hence the case numbers for wheezing group was determined at 70.

### Animals

E3 rats were housed under specific pathogen free condition and fed with standard rodent chow and drinking water *ad libitum*. The rats were 8–12 weeks old when the AIPI model was established. Age- and gender-matched rats were divided into three groups and each group consisted of ten rats. Induction and evaluation of antigen induced pulmonary inflammation (AIPI) were as previously described [23]. The animal experiments were performed in accordance with the guide for the care and use of laboratory animals and approved by the Institutional Animal Ethics Committee of Xi’an Jiaotong University. Rats were anaesthetized by intraperitoneal injection with 30 mg/kg mebumalnatrium, and abdominal aorta blood was drained afterwards. After blood was drained, lungs and spleens were promptly removed. The bronchoalveolar lavage fluid (BALF), plasma, spleens and lavaged lungs were stored at -80 °C. Unfortunately, blood coagulation occurred in three rats from control group and five rats from acute AIPI group during this experiment. Hence, plasma from eight abdominal aorta blood samples of those animals was unavailable for further analysis.

### Details for AIPI rat model

Briefly, E3 rats were immunized by i.p. injection with 1 ml emulsion solution containing 1 mg OVA (Sigma-Aldrich, USA) and 50 mg Al(OH)_3_ (Pierce, USA). Two weeks after immunization, rats were further challenged intranasally with 100 μl OVA solution (1 mg/ml in PBS) for 1 week (acute group) or 8 weeks (chronic group). Rats from control group were sham sensitized and exposed to the same volume of solvent.

Bronchoalveolar lavage fluid (BALF) was collected by gently flushing and withdrawal through the tracheal route with 2 ml PBS for three times (total volume of 6 ml). BALF cell counts were determined by calculating cell pellets from BALF using a hemocytometer. Animal blood was drawn from abdominal aorta for plasma preparation.

### Blood collection and differential cell counting

2 ml blood was collected using citrate-containing vacuum blood drawing tube. Blood was centrifuged at 1 800 g for 20 min at 4 °C to obtain plasma. Plasma was transferred carefully, ensuring that none of the precipitated cell debris pellets were harvested by leaving a sufficient volume of plasma in the tube and preventing any touching of the pipette tip from the interface. Hemocytolysis was avoided since the products might affect miRNA quantification. The blood cells were obtained by mixing the blood cell pellets with saline at one in five volume of total blood. Plasma and blood cell mixture were separated, divided into 200 μl aliquots and stored immediately at -80 °C. Differential cell count was performed using a fully-automatic five-classifying blood cell analyzer (Sysmex XS-800i, Sysmex Corporation).

### RNA isolation and miRNA reverse-transcription

Total RNA from plasma or BALF was isolated using Tri-reagent® BD or LS according to the manufacturer’s instructions (Molecular Research Center). Plasma at volume of 200 μl was prepared in a 1.5 ml RNase free eppendorf tube, mixed with 750 μl Tri-reagent® BD (Molecular Research Center) and 20 μl 5 M HAc, and denatured by vortexing for 1 min. Synthetic cel-miR-39 (GenePharma) of 5 μl 50pM (5nM in blood cell isolation), a single-stranded RNA originated from *Caenorhabditis elegans*, was added as an internal control, leading to a 1.25pM concentration spiked into 200 μl plasma. The sample was set still for 5 min at room temperature, added with 0.2 volume chloroform, followed by vortexing for 30s, and set at room temperature again for 5 min. After centrifugation at 12,000 g at 4 °C for 15 min, upper layer liquid was drawn into another new RNase free tube. Isopropanol of 0.5 volume was added, and the tube was incubated at -20 °C over night. After centrifugation at 12,000 g for 20 min, supernatant was discarded. Ethanol of 1 volume 75 % was added, followed by centrifugation at 7500 g for 15 min at 4 °C. Supernatant was discarded, and remaining pellets were dried for 20 min at room temperature. The faintly visible pellet was dissolved in 8 μl RNase free water. Total RNA in 1 μl was evaluated for concentration and purity using a UV-Vis spectrophotometer (NanoDrop 2000c, Thermo), and 6 μl was used in reverse-transcription (RT) reaction.

RNA concentration and A260/280 ratio of the three groups were compared and were found to be comparable (Additional file 1: Table S3). Total RNA from BALF was isolated following similar procedures using the product of Tri-reagent® LS (Molecular Research Center). Tissue total RNA was isolated using Trizol® (Invitrogen) according to similar procedures without addition of HAc or synthetic miRNA. Total RNA was dissolved in 30 μl RNase free water (1.2 μl for RNA evaluation, 5 μg for RT).

For tissue total RNA isolation, Trizol® (Invitrogen) was used according to similar procedures without addition of synthetic miRNA, and 5 μg tissue total RNA was used for RT reaction. A universal miRNA one-step RT kit (Takara Bio Inc.) was chosen for RT process. Briefly, total RNA was mixed with 10 μl 2 × RT buffer mixture, 2 μl 0.1%BSA, 2 μl RT enzyme mixture and RNase free water to a total volume of 20 μl. Reaction was finished at 37 °C for 60 min, followed with 85 °C for 5 s.

### Real-time quantitative PCR

After preparation by using 80 μl easy dilution buffer from kit, cDNA of 2 μl was used for quantification by mixing with miRNA-specific forward primer (depicted in Additional file 1: Table S4), universal reverse primer, pure water (Milli-Q, Millipore Corporation, Billerica, USA) and 2 × FastStart universial SYBR Green master dye (Roche Applied Science) in iQ™5 system (Bio-Rad Laboratories). Cel-miR-39 or U6 snRNA was amplified respectively in body fluid or tissue samples for normalization. Raw data were analyzed with automatic Ct setting for adapting threshold of Ct determination. For reverse transcription-quantitative polymerase chain reaction (RT-qPCR) of miRNA expression in plasma and BALF, the results are shown as relative expression of each miRNA normalized by spiked-in cel-miR-39 that was automatically calculated by 2^-ΔΔCt^ method [24], and finally processed into fold change of expression. For RT-qPCR of miRNA expression in solid tissue, U6 snRNA were used as internal control.

Several controls were set for each step of real-time quantitative PCR (RT-qPCR). A non-spike-in control reflected any possible detection of signal using primers for foreign spike-in cel-miR-39, a non-template control detected the non-specific amplication, and a non-RT control monitored the contamination from genomic DNA amplification. A weighted mean cDNA sample pooling 1 μl cDNA dilution solution from each of the 140 samples was used as a quality control (QC) between RT-qPCR batches. The QC sample was present in each batch and the individual data of QC sample in various batches offered the reference for correction. For measurement of miRNA expression in solid tissue, the results were shown as relative expression of each miRNA normalized by U6 snRNA automatically calculated by 2^-ΔΔCt^ method [24]. The fold change of expression was further shown with the average data for control group universally set as one. For more detailed information, please refer to Additional file 1.

### Enzyme-linked immunosorbent assay

Human total IgE was detected using an ELISA quantitation set (Bethyl Laboratory, USA, Cat No.E80-108). Briefly, plasma samples (diluted by five times) were captured by a coating antibody, blocked by buffered BSA solution and further bind with detecting antibody conjugated with horseradish peroxidase (HRP). Chromogenic substrate 3, 3’, 5, 5’-tetramethylbenzidine (TMB) substrate was added for reaction, which was stopped by sulfuric acid and finally detected at 450 nm. Series diluted standard protein of human total IgE was used for the four-parameter standard curve. Both the test samples and standard protein were prepared in duplicates. Absolute concentration of total IgE from each plasma sample was hence calculated using the standard curve.

### Statistical analysis

Differences among three groups were analyzed by Kruskal-Wallis test followed Mann-Whitney test for difference between groups according to data characteristics. Correlations were analyzed with Spearman correlation test, and multiple comparisons were applied with Bonferroni adjustment method. The ability of biomarkers to discriminate patients from control individuals was assessed by receiver operating characteristic (ROC) curves. *P* value less than 0.05 was considered statistically significant, but *P* value less than 0.1 was used to select miRNAs for further validation test.

## Results

### MiR-21 and miR-26a significantly increased in plasma from wheezing children

**Fig. 1:**
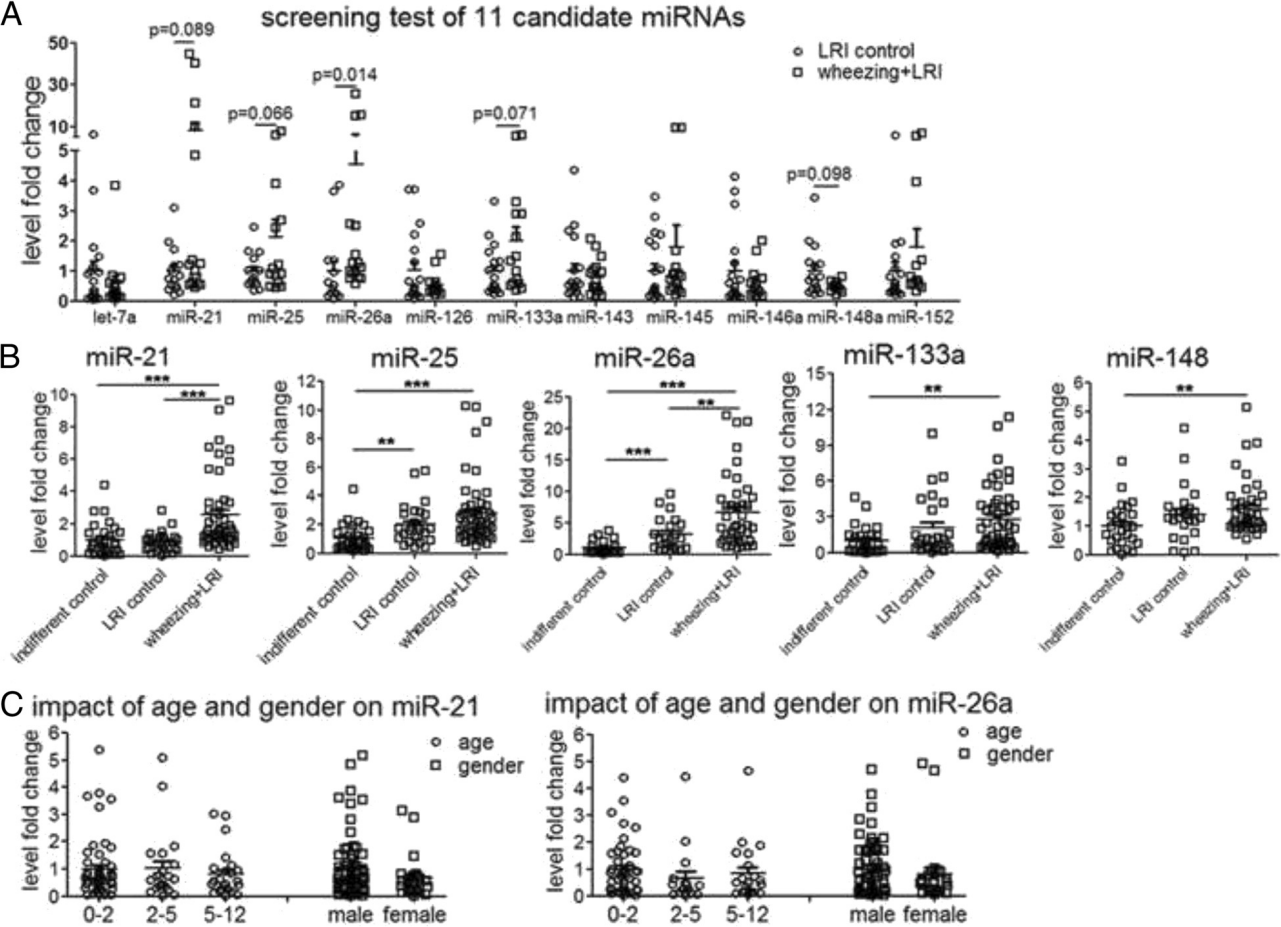
Differential levels of miR-21 and miR-26a in plasma from wheezing children. **a** RT-qPCR results of candidate miRNAs in plasma from 20 LRI control and 20 wheezing + LRI children. **b** RT-qPCR results of miR-21, miR-25, miR-26a, miR-133a and miR-148 in plasma from 35 indifferent control, 35 LRI control and 70 wheezing + LRI children. **c** Scattered dot plots of plasma miR-21 and miR-26a in wheezing + LRI children of different ages and genders. Cel-miR-39 was used as internal control for circulating miRNA. Differences among three groups were analyzed by Kruskal-Wallis test followed Mann-Whitney test for difference between groups according to data characteristics. **: *p* < 0.01, ***: *p* < 0.001

Eleven candidate miRNAs identified in previous studies to be important in asthma conditions (Additional file 1: Table S1) were selected in an initial screen for potential plasma biomarkers in pediatric bronchial asthma. In a set of wheezing + LRI patients (*n* = 20) and age- and gender-matched LRI control children (*n* = 20), miR-21, miR-25, miR-26a, miR-133a and miR-148 showed potential statistical differences between the patient and control groups (*p* < 0.10) (Fig. 1a). These miRNAs were further investigated in a cohort of 70 wheezing + LRI children, 35 indifferent control and 35 LRI control. While there is a significant increase in the plasma level of miR-21 and miR-26a in wheezing + LRI patients compared with indifferent control or LRI control (*p* < 0.01), there was no significant difference in the plasma level of miR-25, miR-133a and miR-148 between wheezing + LRI group and LRI control (Fig. 1b). There was no difference in the plasma level of miR-21 and miR-26a between different age groups of wheezing + LRI children, and gender did not influence on plasma level of expression of miR-21 and miR-26a (Fig. 1c).

### Plasma miR-21 and miR-26a were evaluated for its clinical implication in childhood wheezing

**Fig. 2:**
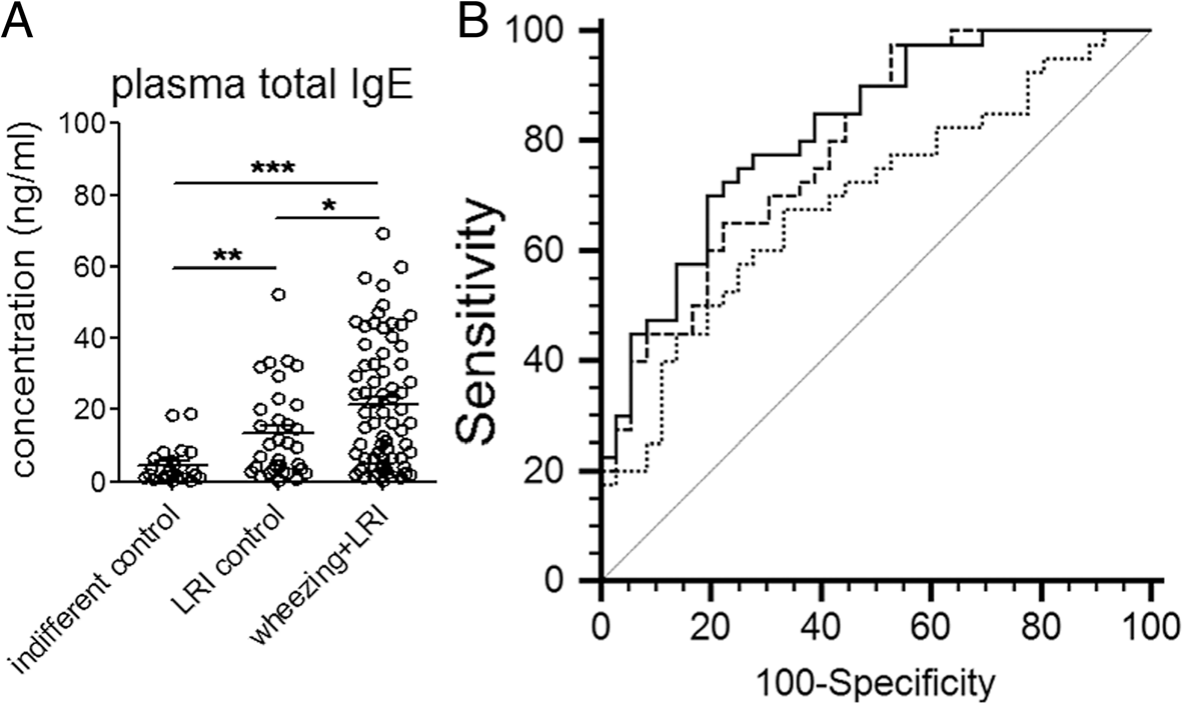
Clinical implication of plasma miR-21 and miR-26a in childhood wheezing. **a** Total IgE in plasma detected using ELISA method. **b** ROC curves of plasma total IgE, plasma miR-21 and miR-26a for differentiation of childhood wheezing. Differences among three groups were analyzed by Kruskal-Wallis test followed Mann-Whitney test for difference between groups according to data characteristics. *: *p* < 0.05, **: *p* < 0.01, ***: *p* < 0.001

Since plasma total IgE is an important biomarker in pediatric asthma, we determined plasma total IgE level in the wheezing + LRI patients, indifferent control and LRI control groups by ELISA. Our data showed that there was a significant increase in plasma total IgE in wheezing + LRI patients compared with indifferent control (*p* < 0.001) or LRI control (*p* < 0.01) group (Fig. 2a). ROC plots of plasma miR-21, miR-26a and total IgE as the potential wheezing index were obtained using MedCalc software. Area under curve (AUC) reflects preferable index for variable separation, and our data showed that plasma miR-21 (AUC of 0.802, *p* < 0.001) was more preferably than plasma miR-26a (AUC = 0.769, *p* < 0.001), and plasma total IgE (AUC = 0.688, *p* < 0.01) (Fig. 2b). AUC values for the combination of plasma total IgE and plasma miR-21, as well as the combination of plasma total IgE, plasma miR-21 and miR-26a were 0.814 and 0.830 respectively.

### Content of circulating miR-21 and miR-26a was not correlated with the corresponding intracellular expression or various cell counts in blood

**Fig. 3:**
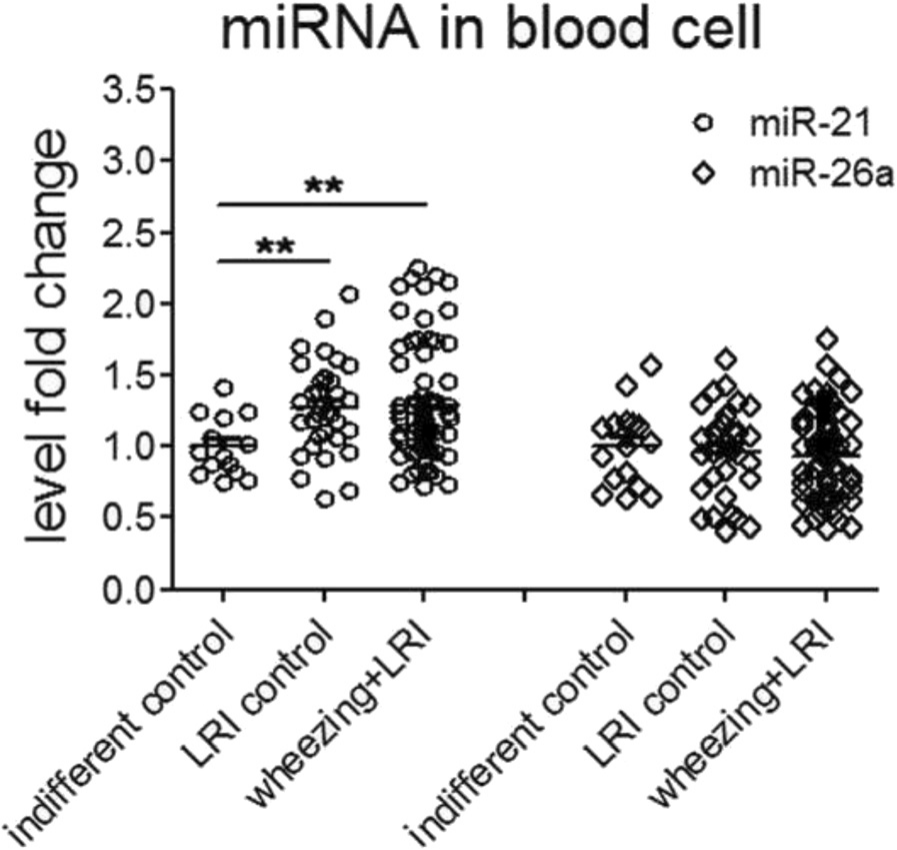
MiR-21 and miR-26a expression in blood of wheezing children. MiR-21 and miR-26a in blood cells from 19 indifferent control, 35 LRI control and 70 wheezing + LRI children were detected using RT-qPCR. Cel-miR-39 was used as internal control for circulating miRNA detection. Differences among three groups were analyzed by Kruskal-Wallis test followed Mann-Whitney test for difference between groups according to data characteristics. *: *p* < 0.05, **: *p* < 0.01, ***: *p* < 0.001

In order to investigate if the increase in plasma miRNAs was derived from peripheral blood cells, we determined the levels of miR-21 and miR-26a in blood cell pellets from corresponding individuals of validation samples (Fig. 3). MiR-21 level was comparable between LRI control and asthma + LRI groups. There was also no difference in the expression of miR-26a in blood cells between any groups of the validation samples (Additional file 1: Figure S2). Correlation analyses were performed between plasma miR-21 (miR-26a) and 14 various indexes for blood components including total leukocytes and platelet numbers, absolute and relative cell counts of monocytes, neutrophils, basophils, lymphocytes, eosinophils and miR-21/miR-26a (Additional file 1: Table S5). The results showed that plasma level of both miR-21 and miR-26a were not correlated with those blood components.

### Increasing miR-21 and miR-26a were found in plasma and bronchoalveolar lavage fluid of AIPI rats

In order to determine whether circulating miR-21 and miR-26a change in the development of airway inflammation, we studied the level of expression of miR-21 and miR-26a in rats with antigen-induced pulmonary inflammation.

**Fig. 4:**
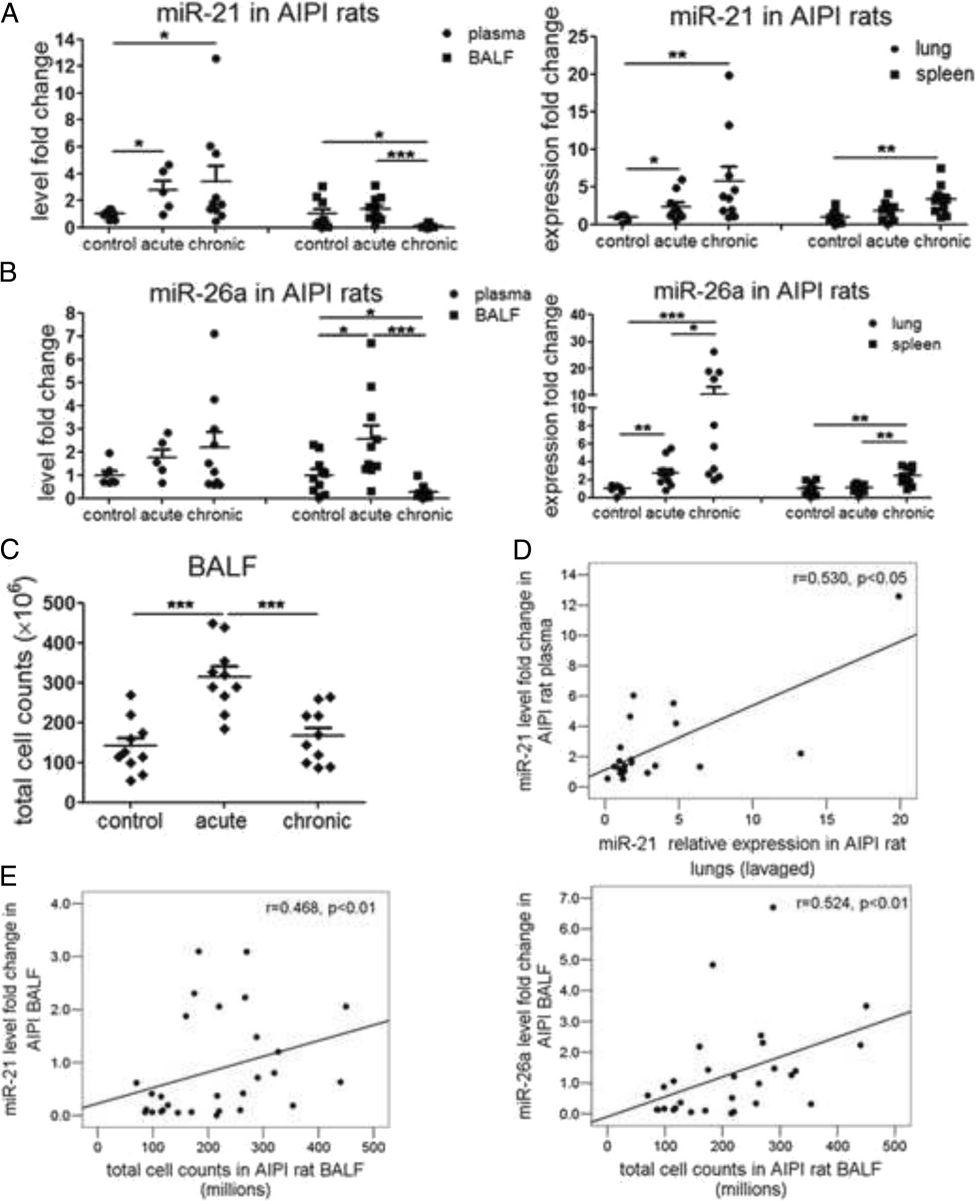
MiR-21 and miR-26a level in body fluids and solid organs from rats with antigen induced pulmonary inflammation. **a**-**b** miR-21 (**a**) and miR-26a (**b**) in plasma, BALF, spleens and lavaged lungs of rats from control, acute and chronic AIPI rats. **c** Total cell counts in BALF of AIPI rats. **d** Scatter dot plots of correlation between plasma and lung miR-21. **e** Scatter dot plots of correlation between BALF miR-21 (miR-26a) and BALF cell counts. BALF stands for bronchoalveolar lavage fluid. RT-qPCR was used to determine the miRNA levels. Cel-miR-39 and U6 snRNA were used as internal controls for circulating or intrinsic miRNA detection. Data were analyzed by Kruskal-Wallis test followed with Mann-Whitney test for analysis between groups. Spearman correlation method was used for correlation analysis. *: *p* < 0.05, **: *p* < 0.01, ***: *p* < 0.001

The acute and chronic AIPI was induced in inbred E3 rats characterized by the extensive inflammation and pathological lung remodeling. In acute AIPI rats, we observed that there was a significant increase in the total cell counts in BALF (*p* < 0.001), indicating the infiltration of inflammatory cells into alveolus (Fig. 4c). In the acute AIPI group, miR-21 level increased in plasma and lungs (both *p* < 0.05) compared with that of the control group, but remained unchanged in BALF and spleen. MiR-26a expression of the acute AIPI rats increased in BALF (*p* < 0.05) and lungs (2.8 fold increase, *p* < 0.01) compared with that of the control group, but remained unchanged in plasma and spleen. In chronic AIPI rats, the number of infiltrated inflammatory cells in alveolus is comparable with that of the control rats. The miR-21 expression increased in plasma (*p* < 0.05), lungs (5.7 fold increase, *p* < 0.01) and spleens (3.3 fold increase, *p* < 0.01) in the chronic AIPI rats compared with that of the control group. Both the miR-21 and miR-26a levels sharply decreased in chronic AIPI rat BALF. While there was no change in the expression level of miR-26a in plasma, the expression level of miR-26a was higher in lungs (10.5 fold increase, *p* < 0.001) and spleens (2.4 fold increase, *p* < 0.01) in the chronic group compared with that of control group, and also compared with that of acute group (*p* < 0.05 for lungs, *p* < 0.01 for spleens). Correlation test showed that miR-21 expression in AIPI rat plasma was positively correlated with its relative expression in lavaged lungs (r = 0.530, *p* < 0.05) (Fig. 4d). However, the expression of miR-26a in the plasma did not correlate with the expression of miR-26a in the lung and spleen. In addition, BALF miR-21 (r = 0.468, *p* < 0.01) and miR-26a (r = 0.524, *p* < 0.01) were both highly positively correlated with BALF cell counts (Fig. 4e-f).

## Discussion

### Our findings

Firstly, the increase of plasma miR-21 and miR-26a was screened out and validated in wheezing children. The expression level of both plasma miRNAs does not vary between different gender and age groups. Secondly, ROC results showed that plasma miR-21 was more preferable to plasma miR-26a and plasma total IgE as biomarker, and were of potential clinical significance. In addition, plasma miR-21 and miR-26a levels did not correlate with total IgE, various counts of different types of leukocyte or miRNA expression in corresponding blood cells. In rats with acute and chronic pulmonary inflammation, miR-21 levels increased in plasma and lung compared with control rats. Circulating miR-21 and miR-26a in BALF were strongly correlated with the infiltrated inflammatory cell numbers.

### Connections between previous studies and our findings

Previous studies have shown that miR-21 participates in various molecular events important for inflammatory and remodeling process of pulmonary cells [11, 25–27]. However, little is known about miR-26a in such process, and both miRNAs hardly intersected with each other during inflammation. It is known that miR-26a and miR-21 are induced during mechanical stretch in human smooth muscle cells [14, 28]. These findings on intracellular miR-21 and miR-26a combined with our findings on extracellular ones might provide more clues on their potential as indicators for lung inflammation.

### Strength of the study

While it has been suggested that extracellular miRNAs should be further studied for a better understanding of asthma mechanism and diagnosis [29], there has so far been no report focusing on extracellular miRNAs in adult or pediatric asthma, and in wheezing children. In the present study, we showed that plasma miR-21 and miR-26a are strongly implicated in childhood wheezing and airway inflammation.

BALF, the body fluid which are applicable for proteomic investigations, has aroused attention as altervative non-invasive approach for asthma diagnosis [30]. Our results also suggested that miRNAs in BALF display potential as biomarkers of airway inflammation in future. The positive correlation between BALF miR-21 or miR-26a expressions and BALF cell count indicated that the cell-free miR-21 or miR-26a may originate from infiltrated inflammatory cells in airways.

From our data, it is shown that in plasma from both wheezing children and rats with pulmonary inflammation, symptomatic individuals have overlap levels compared with control group. However, it is still very clear that some of the symptomatic individuals have high level than mean + 2SEM of control values. Hence, in this situation, plasma miR-21 and miR-26a can be used to identify some recurrent wheezing children with high asthma risk.

### Limitations

We started with 11 candidate miRNAs which were previously reported to be strongly implicated in house dust mite or ovalbumin antigen induced asthmatic mice and cytokine-stimulated human primary airway cells. Of course, we may have missed out miRNAs which are involved in children with recurrent wheezing but not reported in previous studies. Based on our discoveries, we hope that future studies could further investigate the clinical application of circulating miRNA in pediatric asthma diagnosis with more samples and more global assays.

We did not assess sensitivity and specificity with an optimal cut off concentration for these circulating miRNAs just like many other potential biomarkers. This is because the circulating miRNA was detected by using RT-qPCR, which is a relative quantitative method for RNA level. We tried to optimize the method using synthesized miRNAs as calibrators. However, these calibrator miRNAs should go through RNA isolation, reverse transcription and qPCR all together to calculate the absolute concentration in each sample. In particular, the reverse transcription process is a complicated thermo kinetic reaction, which makes the absolute quantitative PCR data for RNA level more unstable compared with DNA copy number detection. Therefore, like other scientists, we chose to evaluate the clinical potential and implication of circulating miRNAs in various disease conditions by detecting the relative expression of miRNAs. Data are shown as fold change against the average value of the control group. While we concluded that the level of expression of these 2 circulating miRNAs increased in plasma from wheezing children, we did not recommend cut-off level for daily clinical practice.

## Conclusions

In summary, this study demonstrated the increase of circulating miR-21 and miR-26a and their clinical potential in pediatric recurrent wheezing, and also found that these circulating miRNAs increased in plasma and bronchoalveolar lavage fluid from animals under inflammatory stress.

## Additional file

Additional information are arranged together in the single additional PDF file includes several contents, listed as follows. Additional file 1: Table S1. miRNAs participating in asthmatic condition. **Table S2**: the theoretical minimum sample volume calculated according to results from small sample test. **Table S3**: RNA concentration and purity validation during the RNA isolation process (quantitative data are shown as mean ± SEM). **Table S4**: primer information of miRNAs. Table S5: correlation analysis of plasma miR-21 or miR-26a and various leukocyte counts. **Figure S1**: balanced age distribution of individuals in samples for miRNA screening or validation. **Figure S2**: cell counts in blood of wheezing children. (PDF 1352 kb)
