# Supplementary material for: Extracellular microRNA-21 and microRNA-26a increase in body fluids from rats with antigen induced pulmonary inflammation and children with recurrent wheezing
Source: BMC Pulm Med. 2016 Apr 14;16:50. doi: 10.1186/s12890-016-0216-2 (PMC4831091; doi:10.1186/s12890-016-0216-2)
Supplement: Additional file 1: Table S1. — miRNAs participating in asthmatic condition. Table S2: the theoretical minimum sample volume calculated according to results from small sample test. Table S3: RNA concentration and purity validation during the RNA isolation process (quantitative data are shown as mean ± SEM). Table S4: primer information of miRNAs. Table S5: correlation analysis of plasma miR-21 or miR-26a and various leukocyte counts. Figure S1: balanced age distribution of individuals in samples for miRNA screening or validation. Figure S2: cell counts in blood of wheezing children. (PDF 1352 kb) [file 12890_2016_216_MOESM1_ESM.pdf]

Additional file 1

Evaluation of methodology for circulating miRNA RT-qPCR

Firstly, we examined the specificity of our RT-qPCR system by checking performance in control samples. The spiked in cel-miR-39 was replaced with pure water in non-spike-in control, and the amplification signal of cel-miR-39 was undetectable with a disorganized melting curve， suggesting that cel-miR-39 was not present in human samples and was appropriate as an endogenous control. RT enzyme was replaced with pure water during cDNA processing in non-RT control, and the signals from cel-miR-39 or other endogenous miRNAs were all undetectable. cDNA template was replaced with pure water during qPCR in non-template control, and the signal were absent for all target miRNAs. The results ruled out the presence of non-specific amplification. In addition, to monitor the reproducibility of the plasma miRNA levels, a homemade QC sample was set in each batch of RT-qPCR runs. During four batches of plasma miRNA determination, the raw Ct value of QC sample for cel-miR-39 was 23.59±0.08, with a CV of 0.68%, and for target miRNA (such as miR-21) the resultwas 22.34±0.014,with a CV of 0.13%. All above-mentioned results demonstrated the reliability of this methodology.

Cell counts in plasma from wheezing children

According to the results from routine blood test (Figure S2), LRI condition could increase absolute and relative neutrophil cell counts, relative basophil cell counts and absolute platelet numbers, as well as decrease absolute and relative lymphocyte counts. These various leukocyte counts could respond to infection and

inflammation to some extent; however, none of these cell counts could effectively indicate wheezing condition.

Table S1.miRNAs participating in asthmatic condition

miRNA	expression pattern and potential function	validated targets in airway inflammation	references
let-7a	decreased in Th2 splenic cells of asthmatic mice	IL-13	1
miR-21	upregulated in allergic airway inflammation, LPS treated RAW264.7 and during macrophage-like differentiation process	IL-12p35 & PDCD4	2, 3
miR-25	regulating the level of many extracellular matrix proteins and contractile proteins in cytokine-stimulated human airway smooth muscle cells (HASMC)	KLF4	4
miR-26a	induced in HASMC by mechanical stretch;<br>inducing human airway smooth muscle hypertrophy by suppressing GSK-3β	No data	5
miR-126	induced in airway wall tissue of chronically challenged mice;<br>participating in the recruitment of intraepithelial eosinophils and regulation of the effector function of Th2 cells	PU.1	3, 6
miR-133a	decreasedin bronchialsmooth muscle of the challengedmice and negatively regulating RhoA	No data	7
miR-143 and miR-672	induced in pathological lung remodeling accompanied with the decrease of MMP12	No data	8
miR-145	participating in house dust mite-induced allergic airway disease in mice by regulating IL-5, IL-13	No data	9
miR-146a	modulating human bronchial epithelial cell survival through up-regulating Bcl-xl and STAT3 phosphorylation which might contribute the tissue repair and remodeling;<br>inhibiting IL-1β induced IL-8 and RANTES releasein human lung alveolar epithelial cell	No data	10, 11
miR-148 and miR-152	differentially targeting the specific HLA-G SNP alleles in human asthma	HLA-G	12

References in Table S1

1. Polikepahad S, Knight JM, Naghavi AO, Oplt T, Creighton CJ, Shaw C, et al. Proinflammatory role for let-7 microRNAS in experimental asthma. J Biol Chem 2010; 285:30139-49.
2. Lu TX, Munitz A, Rothenberg ME. MicroRNA-21 is up-regulated in allergic airway inflammation and regulates IL-12p35 expression. J Immunol 2009; 182:4994-5002.
3. Mattes J, Collison A, Plank M, Phipps S, Foster PS. Antagonism of microRNA-126 suppresses the effector function of TH2 cells and the development of allergic airways disease. Proc Natl Acad Sci U S A 2009; 106:18704-9.
4. Kuhn AR, Schlauch K, Lao R, Halayko AJ, Gerthoffer WT, Singer CA. MicroRNA expression in human airway smooth muscle cells: role of miR-25 in regulation of airway smooth muscle phenotype. Am J Respir Cell Mol Biol 2010; 42:506-13.
5. Mohamed JS, Lopez MA, Boriek AM. Mechanical stretch up-regulates microRNA-26a and induces human airway smooth muscle hypertrophy by suppressing glycogen synthase kinase-3beta. J Biol Chem 2010; 285:29336-47.

6. Collison A, Herbert C, Siegle JS, Mattes J, Foster PS, Kumar RK. Altered expression of microRNA in the airway wall in chronic asthma: miR-126 as a potential therapeutic target. BMC Pulm Med 2011; 11:29.
7. Chiba Y, Tanabe M, Goto K, Sakai H, Misawa M. Down-regulation of miR-133a contributes to up-regulation of Rhoa in bronchial smooth muscle cells. Am J Respir Crit Care Med 2009; 180:713-9.
8. Garbacki N, Di Valentin E, Piette J, Cataldo D, Crahay C, Colige A. Matrix metalloproteinase 12 silencing: a therapeutic approach to treat pathological lung tissue remodeling? Pulm Pharmacol Ther 2009; 22:267-78.
9. Collison A, Mattes J, Plank M, Foster PS. Inhibition of house dust mite-induced allergic airways disease by antagonism of microRNA-145 is comparable to glucocorticoid treatment. J Allergy Clin Immunol 2011; 128:160-7 e4.
10. Liu X, Nelson A, Wang X, Kanaji N, Kim M, Sato T, et al. MicroRNA-146a modulates human bronchial epithelial cell survival in response to the cytokine-induced apoptosis. Biochem Biophys Res Commun 2009; 380:177-82.
11. Perry MM, Moschos SA, Williams AE, Shepherd NJ, Larner-Svensson HM, Lindsay MA. Rapid changes in microRNA-146a expression negatively regulate the IL-1beta-induced inflammatory response in human lung alveolar epithelial cells. J Immunol 2008; 180:5689-98.
12. Tan Z, Randall G, Fan J, Camoretti-Mercado B, Brockman-Schneider R, Pan L, et al. Allele-specific targeting of microRNAs to HLA-G and risk of asthma. Am J Hum Genet 2007; 81:829-34.

Table S2.The theoretical minimum sample volume calculated according to results from small sample test

miRNAs	LRI control group (n=20)		wheezing+LRI group (n=20)		theoretical minimum sample volume#
	mean	SD	mean	SD	
miR-21	1.00	0.78	8.20	14.53	64
miR-25	1.00	0.60	2.19	2.22	55
miR-26a	1.00	1.24	4.53	7.40	69
miR-133	1.00	0.83	2.00	1.86	54
miR-148	1.00	0.85	0.48	0.18	42

#: Theoretical minimum sample volume was calculated using the statistical formula $n = \frac{2\sigma^2 (t_\alpha + t_\beta)^2}{(\mu_1 - \mu_2)^2}$, in which α=0.05, β=0.2, t_α=1.96, t_β=0.842

Table S3. RNA concentration and purity validation during the RNA isolation process (quantitative data are shown as mean±SEM)

groups	n	RNA concentration (ng/µl)	A_{260}/A_{280} ratio
indifferent control	35	389±17	1.731±0.018
LRI control	35	402±33	1.684±0.023
wheezing+LRI	70	381±26	1.722±0.023

Table S4.Primer information of miRNAs

miRNA name	accession No.	primer sequences
cel-miR-39-3p	MIMAT0000010	TCACCGGGTGTAAATCAGCTTG
hsa-let-7a-5p	MIMAT0000062	TGAGGTAGTAGGTTGTATAGTT
hsa(rno)-miR-21	MIMAT0000076	TAGCTTATCAGACTGATGTTGA
hsa-miR-25-3p	MIMAT0000081	CATTGCACTTGTCTCGGTCTGA
hsa(rno)-miR-26a-5p	MIMAT0000082	TTCAAGTAATCCAGGATAGGCT
hsa-miR-126-3p	MIMAT0000445	TCGTACCGTGAGTAATAATGCG
hsa-miR-133a	MIMAT0000427	TTTGGTCCCCTTCAACCAGCTG
hsa-miR-143-3p	MIMAT0000435	TGAGATGAAGCACTGTAGCTC
hsa-miR-145-5p	MIMAT0000437	GTCCAGTTTTCCCAGGAATCCCT
hsa-miR-146a	MIMAT0000449	TGAGAACTGAATTCCATGGGTT
hsa-miR-148a-3p	MIMAT0000243	TCAGTGCACTACAGAACTTTGT
hsa-miR-152	MIMAT0000438	TCAGTGCATGACAGAACTTGG
U6 snRNA	GenBank:K00784	F: CTCGCTTCGGCAGCACA<br>R: AACGCTTCACGAATTTGCGT

Note: miRNA accession numbers are obtained from miRBase database (Release 21).

Table S5. Correlation analysis of plasma miR-21 or miR-26a and various leukocyte counts

variable A	variable B	correlation coefficient (*r*)	Significance (*P*)
plasma miR-21	miR-21 in blood cell pellets	0.027	0.810
	plasma total IgE	0.242	0.023
	total leukocyte count	-0.139	0.180
	absolute neutrophil count	-0.053	0.612
	absolute lymphocyte count	-0.127	0.227
	absolute monocyte count	0.008	0.940
	absolute eosinophil count	0.021	0.840
	absolute basophil count	0.112	0.285
	relative neutrophil percentage	0.102	0.333
	relative lymphocyte percentage	-0.145	0.167
	relative monocyte percentage	0.081	0.445
	relative eosinophil percentage	0.095	0.365
	relative basophil percentage	0.030	0.777
	platelet count	-0.019	0.857
plasma miR-26a	miR-26a in blood cell pellets	-0.132	0.270
	plasma total IgE	0.267	0.020
	total leukocyte count	-0.084	0.458
	absolute neutrophil count	0.090	0.427
	absolute lymphocyte count	-0.218	0.054
	absolute monocyte count	0.034	0.768
	absolute eosinophil count	0.099	0.383
	absolute basophil count	0.278	0.013
	relative neutrophil percentage	0.173	0.127
	relative lymphocyte percentage	-0.161	0.157
	relative monocyte percentage	0.137	0.233
	relative eosinophil percentage	0.065	0.570
	relative basophil percentage	0.293	0.009
	platelet count	0.162	0.152

The correlation analyses were analyzed with Spearman correlation with Bonferroni adjustment for multiple comparisons. The corresponding p value was considered as significant if $p<0.0036$ (0.05 was divided by 14).

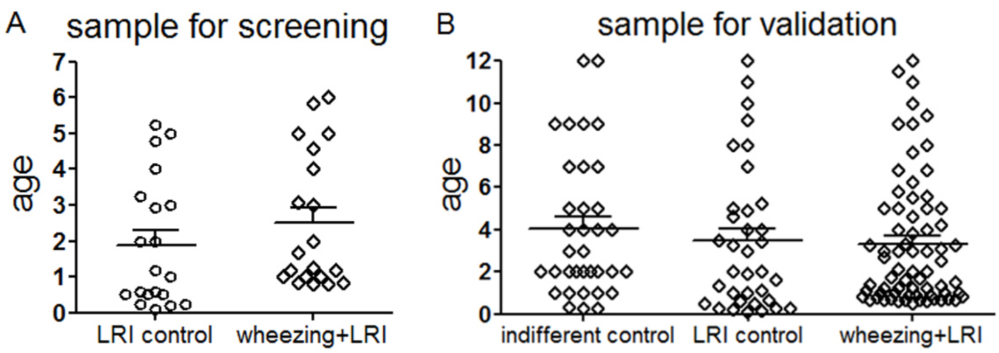

Figure S1 Balanced age distribution of individuals in samples for miRNA screening (a) or validation (b)

Balance of patients' ages in screening sample was analyzed using Mann-Whitney test, and ages in validation sample were analyzed by Kruskal-Wallis test followed with Mann-Whitney test.

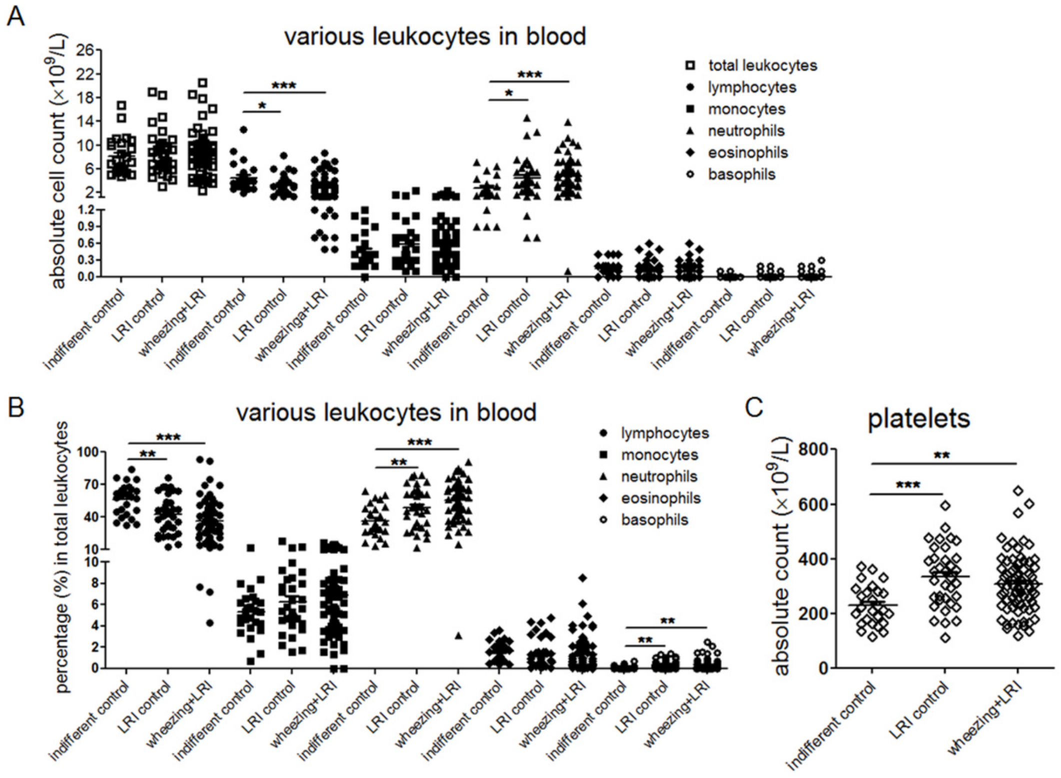

Figure S2 cell counts in blood of wheezing children

A. Absolute cell count of various leukocytes in blood

B. Relative percentage of various cells in total blood leukocytes

C. Platelet numbers in blood

Differences among three groups were analyzed by Kruskal-Wallis test followed Mann-Whitney test for difference between groups according to data characteristics. *: $p<0.05$, **: $p<0.01$, ***: $p<0.001$
